# Efficacy and safety of Zihua Wenfei granules in treatment of postinfectious cough (wind-cold invading lungs syndrome): study protocol for a randomized controlled trial

**DOI:** 10.1186/s13063-020-04487-9

**Published:** 2020-06-19

**Authors:** Huanan Wang, Bin She, Bing Mao, Hongli Jiang

**Affiliations:** Department of Integrated Traditional Chinese and Western Medicine, West China Hospital, Sichuan University, 37 Guoxue Lane, Chengdu, 610041 Sichuan Province China

**Keywords:** Postinfectious cough, Zihua Wenfei granule, Chinese herbal medicine, Randomized controlled trial, Study protocol

## Abstract

**Background:**

Postinfectious cough usually develops and persists following respiratory tract infection. The protracted cough is embarrassing and troublesome and significantly impairs daily life. However, the optimal treatment available for this condition is still not known. This study aims to investigate the efficacy and safety of a new Chinese herbal prescription, Zihua Wenfei granule (ZHWFG), in treatment of postinfectious cough (wind-cold invading lungs syndrome).

**Methods:**

This study is a prospective, multi-center, randomized, double-blinded, parallel group, placebo-controlled trial. A total of 216 adult participants with postinfectious cough will be enrolled from six study centers across China. All participants are randomly allocated to one of three parallel treatment groups: (1) 15 g of active ZHWFG three times daily, (2) 10 g of active ZHWFG plus 5 g of ZHWFG-matched placebo three times daily, and (3) 15 g of ZHWFG-matched placebo three times daily. The treatment duration is 14 consecutive days. The primary outcomes are cough resolution rate and cough relief rate. Secondary outcomes include time to cough resolution, time to cough relief, change from baseline in cough symptom score, cough visual analog scale value, traditional Chinese medicine syndrome score at days 7 and 14, and change of CQLQ from baseline to post-treatment as well as adverse events.

**Discussion:**

This trial may not only investigate the efficacy and safety of ZHWFG in the management of postinfectious cough (wind-cold invading lungs syndrome), but also add the evidence of Chinese herbal medicine in treatment of postinfectious cough and provide an alternative option for the management of postinfectious cough.

**Trial registration:**

ChiCTR1900022078. Registered on 23 March 2019. http://www.chictr.org.cn/showproj.aspx?proj=36547.

## Background

Postinfectious cough is usually categorized as subacute cough lasting for 3 to 8 weeks and is the most common cause of subacute cough responsible for approximate 40 to 50% cases [1, 2]. Postinfectious cough usually develops and persists following respiratory tract infection, accounting for 10 to 40% cases [3, 4].

Postinfectious cough is generally self-limiting and benign; however, the protracted cough may become embarrassing and troublesome, even impairing daily life. In this case, patients may seek for medication therapy. Antihistamines, inhaled or oral corticosteroids, and central acting antitussive agents were used to relieve the cough, but treatment failure during tapering, relapse after drug withdrawal, and potential side effects may be faced [2]. Montelukast, a cysteinyl leukotriene receptor antagonist, was also found to be ineffective in this setting [5]. Inhaled ipratropium and salbutamol may reduce the cough, but the effect was short-acting [6]. Therefore, a growing number of patients turn to complementary and alternative medicine for relieve of cough.

Postinfectious cough is generally defined as exogenous cough in traditional Chinese medicine (TCM). Based on clinical practice and TCM syndrome research, wind-cold invading lungs is the most common syndrome in postinfectious cough [7]. This syndrome is primarily characterized by cough, throat itchiness, a small amount of white phlegm, and chest tightness. The general TCM therapeutic principle is to clear away wind-cold and relieve cough and to warm the lungs and reduce the phlegm.

Zihua Wenfei granule (ZHWFG) is a new Chinese herbal prescription and manufactured by Yuekang Pharmaceutical Co., Ltd (Beijing, China). Table 1 lists the detailed formula. Pre-clinical pharmacodynamic experiments (unpublished data) demonstrated that it can relieve cough and wheeze and reduce phlegm, and it also had antiallergic, anti-inflammatory, and antibacterial effects. A pilot clinical trial showed that Zihua Wenfei Zhisou decoction could alleviate the cough and was safe in treatment of postinfectious cough (wind-cold invading lungs syndrome) [8].

**Table 1:** Formula of Zihua Wenfei granule

Pinyin name	Latin name	Family name
Ziwan	*Aster tataricus L. f.*	Compositae
Kuandong	*Tussilago farfara L.*	Compositae
Shegan	*Belamcanda chinensis L.*	Iridaceae
GanJiang	*Zingiber officinale Rosc.*	Zingiberaceae
Mangguohe	*Mangifera indica L.*	Anacardiaceae
Jingjie	*Nepeta cataria L.*	Labiatae

The aim of this trial is to evaluate the efficacy and safety of ZHWFG in patients with postinfectious cough (wind-cold invading lungs syndrome) in a randomized, double-blind, parallel, placebo-controlled trial. In this study, the investigators hypothesize that (1) the proportion of patients whose cough completely resolve will be higher in the treatment groups, (2) the proportion of patients whose cough relieve will be higher in the treatment groups, and (3) ZHWFG will be safe.

## Design

**Fig. 1:**
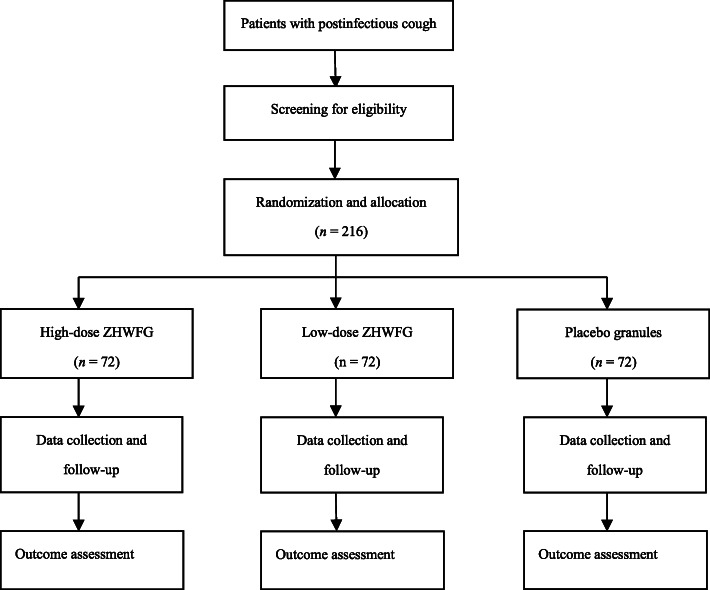
Study flow chart. ZHWFG, Zihua Wenfei Granule

This study is a prospective, multi-center, randomized, double-blinded, parallel group, placebo-controlled trial. The study has been authorized by China State Food and Drug Administration (Approval No. 2016 L05843). In addition, this updated version of trial protocol (version 1.1) has been reviewed and approved by Biomedical Ethics Committee of West China Hospital, Sichuan University (Chengdu, China) on 24 December 2018. Furthermore, this trial was registered with the Chinese Clinical Trial Registry (ChiCTR1900022078) on 23 March 2019. This study is funded by Yuekang Pharmaceutical Co., Ltd (Beijing, China). However, the funder has no input in the study design, future data collection and management, or data analysis and interpretation. A research assistant at each study center is scheduled to recruit patients from late March 2019 to April 2020. The flow chart of the trial is shown in Fig. 1.

### Patient population and setting

In this trial, postinfectious cough is defined as a persistent subacute cough following symptoms of an acute upper respiratory tract infection, lasting for 3 to 8 weeks. Patients with postinfectious cough usually complain of irritable dry cough or with a little mucous sputum and have normal chest radiograph. Patients with persistent subacute cough due to other causes, such as pneumonia, cough variant asthma, upper airway cough syndrome, eosinophilic bronchitis, gastroesophageal reflux cough, asthma, or an acute exacerbation of chronic bronchitis will be excluded [9, 10]. The detailed TCM diagnostic criteria of wind-cold invading lungs syndrome are listed in Table 2. A total of 216 patients of either sex, aged 18–65 years old, will be enrolled from four academic centers and two tertiary hospitals across China: (1) West China Hospital of Sichuan University, (2) the first Affiliated Hospital of Guiyang College of TCM, (3) Affiliated Hospital of Zunyi Medical University, (4) Fujian Province Chinese Medicine Research Institute, (5) Beijing Hospital of TCM, and (6) the first Affiliated Hospital to Henan University of TCM. A research assistant at each participating site takes charge of patient screening and recruiting. The strategies for achieving adequate participant enrollment to reach target sample size include the following: (1) searching patient waiting list from respiratory outpatient, (2) referral from other community hospitals, and (3) enrolling candidate patients through announcement in public. The detailed inclusion and exclusion criteria are presented in Table 3.

**Table 2:** Diagnostic criteria for wind-cold invading lungs syndrome

Category	Symptoms or signs
Main symptom	Cough
Minor symptoms	① Throat itchiness, ② cough aggravated by wind-cold, ③ a small amount of white phlegm, ④ chest tightness
Tongue	Pale red tongue with thin and white coating
Pulse condition	Floating, tight, or normal pulse

Patients with main symptom+①+②+either of the other two minor symptoms+appropriate tongue and pulse condition can be diagnosed as wind-cold invading lungs syndrome

**Table 3:** Patients’ inclusion and exclusion criteria

**Inclusion criteria**	
1. Diagnosis of postinfectious cough	
2. Wind-cold invading lungs syndrome in traditional Chinese medicine Zheng	
3. Cough duration of 3–6 weeks	
4.Baseline cough visual analog scale of 60 mm or more	
5. Aged 18 to 65 years old	
6. Voluntarily provide written and informed consent	
**Exclusion criteria**	
1. Cough caused by asthma, cough variant asthma, upper airway cough syndrome, eosinophilic bronchitis, gastroesophageal reflux cough, or any other concomitant conditions	
2. Patients with severe pulmonary diseases such as lung cancer, lung tuberculosis, or lung fibrosis	
3. Use of an angiotensin-converting-enzyme inhibitor in the last 2 months	
4. Current smokers or recent ex-smokers quitting smoking less than 3 months ago	
5. Patients with temperature of 37.3 °C or above	
6. FeNO > 30 ppb	
7. Bronchial provocation test positive	
8. Post-bronchodilator FEV_1_/FVC < 0.70	
9. Patients with chest X-ray abnormalities	
10. Patient with severe underlying cerebral, hematological, hepatic, or renal disorders or other diseases significantly affecting the survival and prognosis, such as AIDS or cancer	
11. Mental patients or legal disability	
12. ALT or AST > 1.5 times of normal upper limit, urine protein >+, serum creatinine abnormality, white blood cell count < 3 × 10^9^/L or > 10 × 10^9^/L, and neutrophil granulocyte> 80%	
13. Pregnancy or potential pregnancy or lactation	
14. Allergic constitution or known to be allergic to any component in tested drug	
15. Patients taking similar medications in the last 1 month or having participated or participating in another trial in last 3 months	

### Randomization and blinding

Randomization sequence will be created by an independent statistician. Randomization will be carried out in blocks of six using Statistical Analysis System. Participants will be stratified according to study center and randomly assigned in a 1:1:1 ratio. Active and placebo granules will be identical in terms of outer package, labeling, and outer appearance of the medication packs. Random assignment sequence is concealed on a sealed scratch card in each study center to be revealed only when a medical emergency occurs. A research assistant will enroll participants and assign them to interventions. Investigators, participants, and biostatisticians will be masked to treatment assignments during the whole treatment duration. Unblinding is permissible only when a serious adverse event (SAE) or emergency rescue occurs.

### Investigational medication

**Fig. 2:**
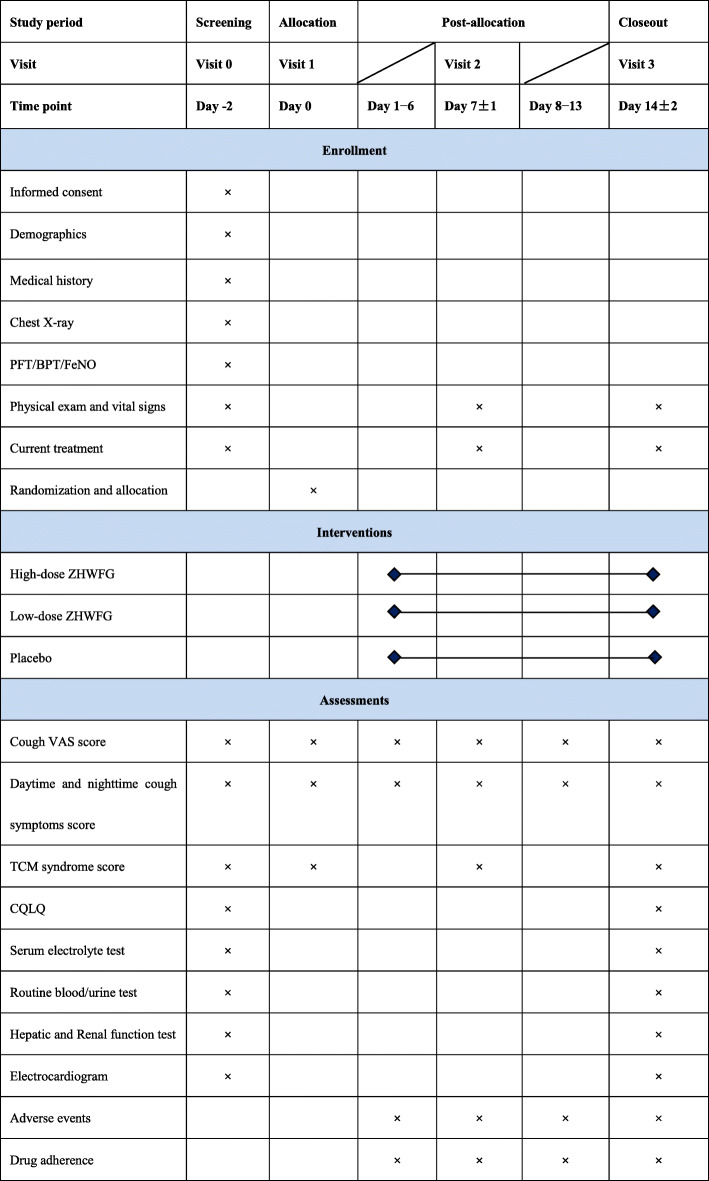
Schedule of study procedures. BPT, bronchial provocation test; CQLQ, cough-specific quality of life questionnaire; FeNO, fractional exhaled nitric oxide; PFT, pulmonary function test; TCM, traditional Chinese medicine; VAS, visual analog scale; ZHWFG, Zihua Wenfei granule

Both active ZHWFG and ZHWFG-matched placebo are manufactured and supplied by Yuekang Pharmaceutical Co., Ltd (Beijing, China) in compliance with the Good Manufacturing Practice. All medications are provided in identical appearance and packaged and labeled in a blinding manner. Each code corresponds to a unique medication assignment number according to randomization list. Each package contains 24 bags of 10 g active ZHWFG or ZHWFG-matched placebo and 24 bags of 5 g active ZHWFG or ZHWFG-matched placebo. The package will be distributed at visits 1 and 2. A clearly visible label on each package states “FOR TRIAL USE ONLY.” The label includes medication information regarding trial approval number, medication number, name, indication and function, dose and specification, dosage regimen, storage requirement, period of validity, and the provider’s name. A specified drug administrator will be independently in charge of receipting, storing, distributing, and retrieving the investigated drugs. All these processes must be recorded in a medication registration book. Figure 2 shows time schedule of enrolment, visits for participants, interventions, and assessments.

### Interventions

Participants will be randomized to orally take one of the following three investigational medications: (1) 15 g of active ZHWFG three times daily (10 g/bag + 5 g/bag), (2) 10 g of active ZHWFG plus 5 g of ZHWFG-matched placebo three times daily, and (3) 15 g of ZHWFG-matched placebo (10 g/bag + 5 g/bag) three times daily. Participants will be required to take the first two bags of medications under observation just after enrollment, and then instructed to take the medications regularly for the next 14 consecutive days.

### Concomitant treatments and forbidden medication

Medications applied to treat the underlying diseases, such as diabetic mellitus and hypertension, are permitted. However, new use of angiotensin-converting-enzyme inhibitors for hypertension is not permitted. Any Western medication or other Chinese medicinal therapy which can relieve cough is prohibited during the study. Additionally, antibiotics are not allowed in this study. If the participants take any forbidden medication, they will be withdrawn. Any medication used in this study must be documented in detail in case report forms (CRFs), including name, dose, dosage regimen, indication, and treatment period.

### Intervention adherence and compliance

Researchers will take the following measures to improve drug adherence: (1) earnestly implementing informed consent regarding the potential benefits and risks, doing comprehensive and detailed explanation works to make the participants fully understand the significance and importance of medication use; (2) recording contact information of participants including landline and mobile phone, email, QQ, and WeChat as detailed as possible to keep them in touch; (3) arranging follow-up visits at their convenient time; and (4) reminding a visit 1 day in advance.

At visits 2 and 3, the specified drug administrator will record the number and detailed specification of medication received, taken, and returned. Medication compliance will be calculated as the actual taken amount of study medication documented in a participant’s daily diary divided by the total amount planned for the participant.

### Withdrawal and termination criteria

Participants may discontinue the study prematurely at any time for any reason. The investigators will be entitled to withdraw any participant because patients develop SAE or allergic reaction, experience condition worsening, loss to follow-up, or take forbidden medications.

The National Pharmaceutical Supervisory and Administrative Department can terminate the whole study for any reason. The investigators may terminate or suspend the study due to SAE, poor efficacy, or significant deviation from the protocol during the study. The sponsors can also terminate the study because of management issues or funding problems.

### Outcome measures

#### Primary outcomes

1. Cough resolution rate: the proportion of patients whose cough is completely resolved.

Cough severity will be self-rated using a validated continuous horizontal 100-mm visual analog scale (VAS), with 0 indicating “no cough” and 100 indicating “worst possible cough” [11]. VAS value of the past 24 h will be evaluated every night at about 8:00 pm during treatment. Complete resolution of cough is defined as a reduction of VAS value to less than 17 mm [12]. Cough resolution rate across groups will be compared at days 7 and 14.

2. Cough relief rate: proportion of patients who achieve cough relief.

Cough relief is defined as a reduction of VAS value by 50% or more from the baseline. Cough relief rate across groups will be compared at days 7 and 14.

#### Secondary outcomes

1. Time to cough resolution: defined as the length of time (day) from treatment start to cough complete resolution.
2. Time to cough relief: defined as the length of time (day) from treatment start to cough relief.
3. Change from baseline in cough symptom score (CSS) at days 7 and 14

CSS is a simple, short, and subjective tool to evaluate the overall frequency and severity of cough in the day and at night. There are daytime (from 8:00 am to 8:00 pm) and nighttime (from 8:00 pm to 8:00 am) CSS, each comprising four possible outcomes with a score range from 0 to 3 points [10]. During the trial, each patient will self-complete and record the CSS in the cough diary every day. Daytime CSS of the last 12 h will be assessed at about 8:00 pm and nighttime CSS of the last 12 h at about 8:00 am. The total CSS is the sum of daytime and nighttime CSS.

4. Change from baseline in TCM syndrome score at days 7 and 14

Each TCM symptom is graded according to TCM symptoms grading criteria (see Additional file 1). Each TCM symptom score of the past 24 h will be evaluated and graded every night at about 8:00 pm during treatment. TCM syndrome score is the sum score of the main and all minor symptoms.

5. Change from baseline in VAS value at days 7 and 14
6. Change of CQLQ from baseline to post-treatment

Cough-specific quality of life questionnaire (CQLQ) is a valid and reliable tool to evaluate the impact of cough on health-related quality of life [11], consisting of six domains including 28 items. The total score ranges from 28 to 112, with lower score indicating a better quality of life [13].

### Adverse events reporting

Adverse events refer to the unexpected medical events that occur during the study, which may not have a causal relationship with the medication. Events can be symptoms, signs, and laboratory abnormalities. Any adverse event will be recorded and reported according to standard procedures. A SAE will be defined as any medical occurrence that results in death, is life-threatening, requires inpatient hospitalization or prolongation of existing hospitalization, results in persistent or significant disability and/or incapacity, is a congenital anomaly or birth defect, or other important medical events. Other events may be considered as a SAE when the event may jeopardize the patient and may require medical or surgical intervention to prevent one of the following outcomes: death, life-threatening, or hospitalization. SAE must be reported to the Biomedical Ethics Committee of West China Hospital of Sichuan University, the sponsor, and China’s State Food and Drug Administration within 24 h.

### Coordination and conduct of the trial

Three investigator meetings have been held to review the study protocol, informed consent, and data collection form. Additionally, before the start of patient recruitment procedures, all documents and funding required for the study and investigational medications should be fully available. One investigator meeting will be held in each participating site to train all trial-related personnel mainly for patient recruitment and data collection. Data monitoring committee is not needed because (1) this is a relatively short-term trial, (2) the primary and secondary endpoints are solely patients’ self-reported symptoms, and (3) the pilot clinical observation did not show potential for toxicity. Interim efficacy analysis will also be not conducted.

The study will be monitored by an independent monitoring committee. The monitor regularly conducts onsite monitoring and reviews the CRFs to ensure participants’ rights and interests, compliance with the trial protocol, source data validity, data accuracy, and data integrity. The study will be audited randomly by an independent auditor regarding trial conduct and compliance with the protocol, standard operating procedures, good clinical practice, and the applicable regulatory requirements.

### Data collection and management

All data including baseline, outcomes, and other trial data will be collected and recorded timely in the paper CRFs. Measures taken to maximize participant retention are as follows: (1) highlighting the significance of participant’s involvement in the study; (2) making use of all available contact information including phone number(s), e-mail address, QQ and WeChat; (3) reminding participants of the upcoming scheduled visits; (4) acknowledging and complimenting participant’s commitment, time, and effort devoted to the study when they complete scheduled visits; (5) attempting to re-contact/re-schedule within 24 h for missed appointments; (6) providing a compensation of ¥400 after completing follow-up; and (7) trying to obtain the participant’s consent for one last visit to collect outcome data when a participant withdraws from the study.

EpiData3.1 database will be used for data entry and management. To ensure the accuracy of the data, double data entry and proofreading will be performed by two independent data administrators. Any query about CRFs will be issued to the investigators and answers should be returned as soon as possible. The database will be backed up regularly and stored off-site. The primary investigators, data administrators, and biostatisticians will check, confirm, and lock the database prior to data processing. Only data administrators and biostatisticians will have access to the final data. Original CRFs and any other records will be kept on file at each participating site for 5 years.

### Sample size calculation

The sample size was calculated on basis of cough relief rate, using PASS 11.0. Based on the pilot clinical observation of 144 patients with postinfectious cough [8], cough relief rate in active treatments was estimated to 70%, with a difference of 30% or more considering clinically significant. Setting a two-sided *α* of 0.05 and *β* of 0.15, the sample size was estimated to be 46 cases for each group. Additionally, this trial involves exploring the effect and safety of different dosage; the sample size for each group was set to 60 cases. Taking approximate dropouts of 20% into account, the number of participants recruited was estimated to be 72 per group. Therefore, a total of 216 patients will be recruited in this trial.

### Statistical analysis

The full analysis set (FAS) includes all randomized participants who take at least one bag of ZHWFG and have follow-up data. If post-treatment follow-up data regarding the primary outcomes are missing, last observation carried forward method will be carried out to adjust for the missing data. Per-protocol set (PPS) includes patients in the FAS who comply with the protocol, are adherent to the assigned study medication, complete the visits and measurements, and have no other protocol violations. Efficacy analysis will be performed based on both FAS and PPS patients. Safety analyses include all randomized participants who take at least one bag of ZHWFG and have data for safety assessment.

An independent statistician will perform statistical analysis according to the statistical analysis plan using Statistical Analysis System (SAS) 9.4. Continuous variables will be expressed as mean (standard deviation) if the data are normally distributed, otherwise median (inter-quartile) alternatively. Categorical variables will be presented as frequency (percentage). Baseline analyses will be performed using analysis of variance or nonparametric tests for continuous variables, and chi-squared or Fisher’s exact tests for categorical variables, respectively. The primary outcomes will be analyzed using CMH-*χ*^2^ test with associated 95% confidence intervals. Time to cough resolution and relief will be analyzed using the Kaplan-Meier approach, and the difference across groups will be compared using the log-rank test. Changes from baseline to post-treatment in CSS, TCM syndrome scores, VAS, and CQLQ will be analyzed using analysis of covariance with associated 95% confidence intervals. A two-sided *P* value of 0.05 or less is considered statistically significant.

### Ethics and dissemination

The study protocol has been reviewed and approved by the Biomedical Ethics Committee of West China Hospital of Sichuan University (Chengdu, China). Any important modifications including the principal investigator, informed consent form, study protocol regarding eligibility criteria, outcomes and analyses, and daily diary card must be resubmitted, reviewed, and approved by the Biomedical Ethics Committee of West China Hospital of Sichuan University (Chengdu, China). Trained investigators will discuss research objective, study procedures, and potential risks and benefits with potential participants and obtain written, informed, and voluntary consent from them. Persons with impaired cognition or communication capacity who might have difficulty understanding information about the study and weighing the risks and benefits are inappropriate to participate in this study. Additionally, pregnant women or women with potential pregnancy or in lactation are excluded from the study. On the consent form, participants will be asked if they agree to use of their data. Participants will also be asked for permission for the research team to share relevant data with people from the study centers taking part in the research or from regulatory authorities, where relevant.

All participants are permitted to discontinue their participation at any time for any reason and are provided continued access to effective care. During the study period, if patients who comply with the trial protocol report any adverse reactions caused by the study medication and need to be treated, the investigators will provide timely and effective treatment freely and arrange close follow-up. Hepatic and renal functions, routine tests of blood and urine, and electrocardiogram will be measured again after treatment to monitor the adverse events. Moreover, each participant will correspond to a unique identity without any other personal identifier to maintain confidentiality. Only investigators, ethical committees, and drug regulation agencies have access to original research data. All biological specimens are exclusively collected for this specified study and will be discarded at the end of the study. This trial does not involve collecting biological specimens for storage.

The full protocol and datasets analyses of the current study are available from the corresponding author on reasonable request. The primary investigators and sponsor will communicate the trial results to participants and all the investigators involved. The trial results will be submitted to national or international conferences or peer-reviewed journals with the sponsor’s permission.

## Discussion

Systematic reviews found that Chinese herbal medicine may alleviate the cough and improve Chinese medicine symptoms and cough-related quality of life [14, 15]. Our recently published randomized controlled trial demonstrated Qingfeng Ganke granule was effective in the treatment of postinfectious cough (wind invading lungs syndrome) [16]. This trial may not only investigate the efficacy and safety of ZHWFG in the management of postinfectious cough (wind-cold invading lungs syndrome), but also add the evidence of Chinese herbal medicine in treatment of postinfectious cough and provide an alternative option for the management of postinfectious cough.

Although postinfectious cough is the most common cause of subacute cough, it is always not easy to rule out other possible causes in diagnosis of postinfectious cough. Besides postinfectious cough, eosinophilic bronchitis (18.5%) and cough variant asthma (14.3%) are among the common causes of subacute cough following acute upper respiratory tract infection [1]. In this trial, bronchial challenge test is used for exclusion of cough variant asthma. Induced sputum analyses test is highly recommended in the establishment of eosinophilic bronchitis; however, the test is limited due to absence of specialized instruments and personnel in primary care setting. A recent systematic review shows that fractional exhaled nitric oxide can predict eosinophilic bronchitis with a sensitivity of 72% and a specificity of 83% respectively [17]. Therefore, in this study, fractional exhaled nitric oxide will be alternatively applied to exclude this condition.

VAS, CSS, and CQLQ will be used to assess the severity, frequency, and impact of cough. Although these measures used in this study are subjective, they can easily be applied to clinical research and practice. Objective markers of cough, such as cough frequency monitors, appear to be valid in assessment of cough frequency; however, poor or moderate responsiveness and inconsistency with other assessments limit their use in research settings [11, 18].

## Trial status

This updated version of trial protocol (version 1.1, 20 November 2018) has been reviewed and approved on 24 December 2018. The first patient was recruited in the trial on 26 March 2019. The recruitment is scheduled to be completed in April 2020.

## Supplementary information

**Additional file 1.** Traditional Chinese medicine symptoms grading criteria

## Data Availability

Not applicable.

## Abbreviations

CQLQ: Cough-specific quality of life questionnaire
CRF: Case report form
CSS: Cough symptom score
FAS: Full analysis set
PP: Per-protocol
RCT: Randomized controlled trial
SAE: Serious adverse event
TCM: Traditional Chinese medicine;
VAS: Visual analog scale
ZHWFG: Zihua Wenfei granule
