# Supplementary material for: Efficacy and safety of Zihua Wenfei granules in treatment of postinfectious cough (wind-cold invading lungs syndrome): study protocol for a randomized controlled trial
Source: Trials. 2020 Jun 19;21:547. doi: 10.1186/s13063-020-04487-9 (PMC7304187; doi:10.1186/s13063-020-04487-9)
Supplement: Supplementary file 1 — Additional file 1. Traditional Chinese medicine symptoms grading criteria [file 13063_2020_4487_MOESM1_ESM.docx]

Supplementary table traditional Chinese medicine symptoms grading criteria

| **Main symptom** | **Score grading** | | | |
| --- | --- | --- | --- | --- |
|  | **0** | **2** | **4** | **6** |
| Cough | Not at all | Occasional cough, or cough  for short periods when falling  asleep | Cough that mildly interfere  with daily activities and sleep | Frequent cough that  seriously interfere with  daily activities and sleep |
| **Minor symptoms** | **Score grading** | | | |
|  | **0** | **1** | **2** | **3** |
| Throat itchiness | Not at all | Mild | Moderate, relief after cough | Sever, not relief after  cough |
| Chest tightness | Not at all | Mild | Moderate | Sever |
| Cough aggravated  by wind-cold | No (0) | | Yes (2) | |
| A small amount of  white phlegm | No (0) | | Yes (2) | |
